# BK_Ca_ channel dysfunction in neurological diseases

**DOI:** 10.3389/fphys.2014.00373

**Published:** 2014-09-29

**Authors:** Prosper N'Gouemo

**Affiliations:** Department of Pediatrics and Interdisciplinary Program in Neuroscience, Georgetown University Medical CenterWashington, DC, USA

**Keywords:** autism, alcohol withdrawal seizures, epilepsy, gain-of-function, loss-of-function

## Abstract

The large conductance, Ca^2+^-activated K^+^ channels (BK_Ca_, K_Ca1.1_) are expressed in various brain neurons where they play important roles in regulating action potential duration, firing frequency and neurotransmitter release. Membrane potential depolarization and rising levels of intracellular Ca^2+^ gated BK_Ca_ channels, which in turn results in an outward K^+^ flux that re/hyperpolarizes the membrane. The sensitivity of BK_Ca_ channels to Ca^2+^ provides an important negative-feedback system for Ca^2+^ entry into brain neurons and suppresses repetitive firing. Thus, BK_Ca_ channel loss-of-function gives rise to neuronal hyperexcitability, which can lead to seizures. Evidence also indicates that BK_Ca_ channels can facilitate high-frequency firing (gain-of-function) in some brain neurons. Interestingly, both gain-of-function and loss-of-function mutations of genes encoding for various BK_Ca_ channel subunits have been associated with the development of neuronal excitability disorders, such as seizure disorders. The role of BK_Ca_ channels in the etiology of some neurological diseases raises the possibility that these channels can be used as molecular targets to prevent and suppress disease phenotypes.

## Bk_Ca_ channels and neuronal excitability

Intrinsic membrane properties play an important role in the control of neuronal activity in the central nervous system (CNS). Alterations of intrinsic membrane properties can contribute to diseases of neuronal excitability such as epilepsy. Potassium (K^+^) channels in particular are well known for their role in the regulation of membrane excitability due to their ability to stabilize the membrane potential. Compelling evidence indicates that K^+^ channels are critical molecular determinants for seizure generation and epileptogenesis. One particular type of K^+^ channel, the large conductance, Ca^2+^-activated K^+^ channel (BK_Ca_, K_Ca1.1_) is considered to be one of the intrinsic molecular determinants for the control of neuronal excitability in the CNS. Unlike other K^+^ channels, BK_Ca_ channels are activated by both voltage and elevated levels of intracellular Ca^2+^, resulting in large K^+^ conductances which in turn re/hyperpolarizes the membrane. The sensitivity of BK_Ca_ channels to Ca^2+^ provides an important negative feedback for Ca^2+^ entry into brain neurons; thus, BK_Ca_ channels may serve as a link between membrane depolarization and Ca^2+^ signaling to provide a rapid response to reduce or prevent neuronal hyperexcitability.

BK_Ca_ channels are tetramers of four α subunits, which form the ion channel pore, and four regulatory β (β 1–4) subunits that are expressed in various tissues, including the brain (Pallanek and Genetzky, 1994; Jiang et al., 1999). BK_Ca_ channels can also be regulated by acidification (Brelidze and Magleby, 2004; Hou et al., 2008), ethanol (Liu et al., 2008), protein kinase phosphorylation (Tian et al., 2001; Zhou et al., 2010), ubiquitination (Liu et al., 2014) and palmitoylation (Shipston, 2013; Zhou et al., 2012). Of particular importance, protein S-palmitoylation (or palmitoylation) and ubiquitination control the cell surface expression and activity of BK_Ca_, thereby critically contributing to BK_Ca_ channel functions (Shipston, 2013; Liu et al., 2014). Notably, the palmitoylation of BK_Ca_ channel β subunits promotes the exit of the pore-forming α subunit from the endoplasmic reticulum and promotes BK_Ca_ channel surface expression (Chen et al., 2013). The BK_Ca_ channel α subunit is encoded by the *Slo*1 gene, which can be subjected to splicing to produce channels with different functional properties and sensitivity to Ca^2+^; including the STREX (stress-axis hormone-regulated exon) channels (Xie and McCobb, 1998; Chen et al., 2005). Expression profiling studies have reported that BK_Ca_ channel α subunits are broadly expressed in the CNS (Chang et al., 1997; Wanner et al., 1999; Sausbier et al., 2006). The regulatory BK_Ca_ channel β 1 and β 4 subunits are also expressed in the brain, whereas the β 2 and β 3 subunits are nearly absent in the brain (Tseng-Crank et al., 1996). BK_Ca_ channels are predominantly located at the axon and presynaptic terminals, associated with glutamatergic synapses in hippocampus and cortex and GABAergic synapses in the cerebellum (Knaus et al., 1996; Hu et al., 2001; Misonou et al., 2006; Martire et al., 2010). These channels are usually found in close proximity to N-methyl-D-asparte receptors (Isaacson and Murphy, 2001) and voltage-gated Ca^2+^ channels (Ca_V_), including Ca_V_1.2, Ca_V_2.2, and Ca_V_2.1 in the CNS (Marrion and Tavalin, 1998; Grunnet and Kaufmann, 2004). During an action potential (AP), both membrane depolarization and elevated intracellular Ca^2+^ can activate BK_Ca_ channels, which in turn contribute to AP fast repolarization, generate the fast component of the afterhyperpolarization (fAHP) and reduce Ca^2+^ influx via inactivation of Ca_V_ channels. Prominently, AP repolarization and fAHP significantly contribute to AP shape and duration. By controlling the AP shape and duration, BK_Ca_ channels can regulate neuronal excitability and some Ca^2+^ transients that underlie the release of neurotransmitter at presynaptic terminals.

**Figure 1:**
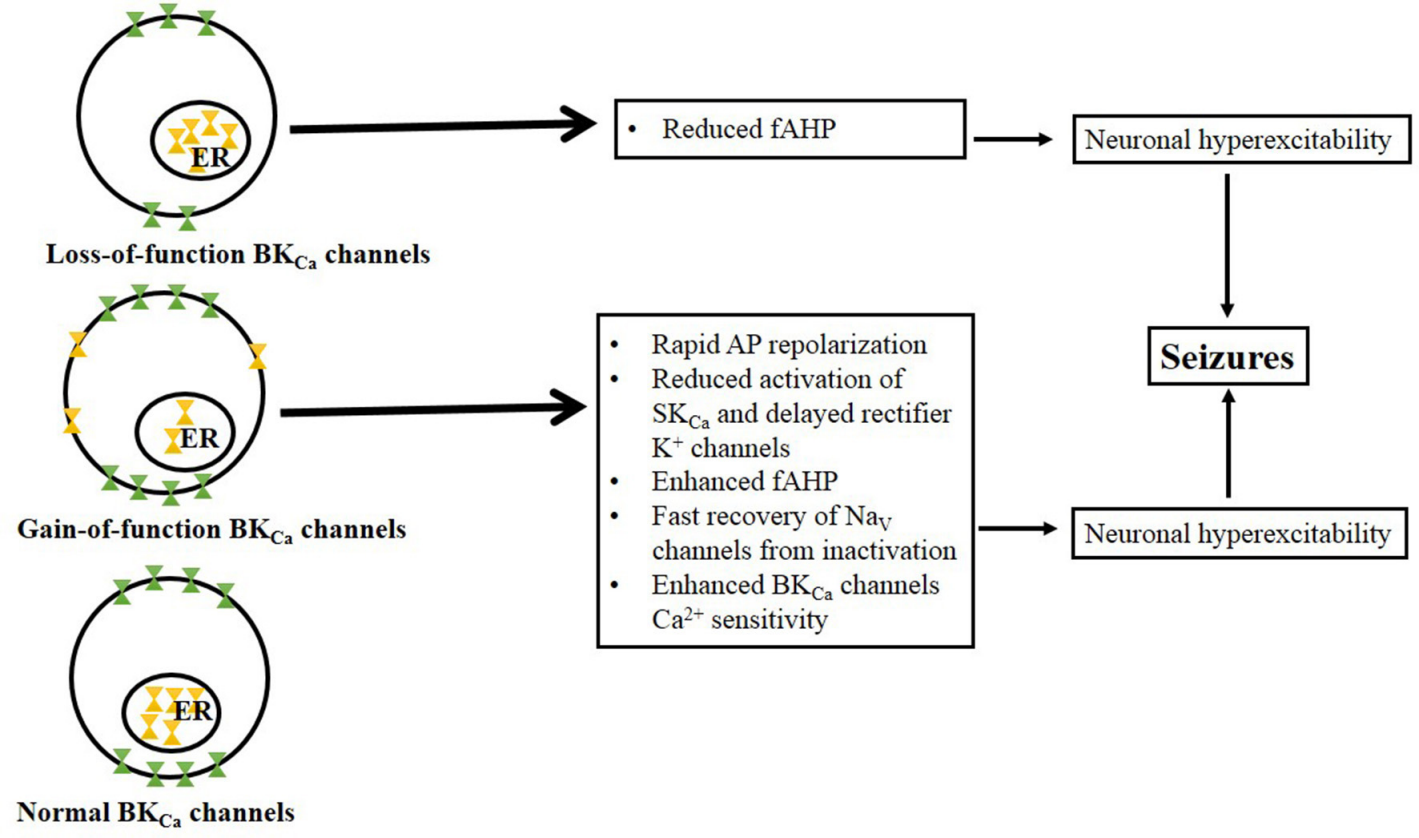
**Proposed mechanisms associated with BK_Ca_ loss-of-function and gain-of-function channels**. BK_Ca_ channel loss-of-function occurs when there is low abundance of the channel at the membrane surface but no change in the BK_Ca_ channel number in the endoplasmic reticulum (ER, note that ubiquitination prevent channels from trafficking to the cell surface). Potential mechanisms underlying neuronal hyperexcitability following BK_Ca_ channels loss-of-function include reduced fAHP conductances. BK_Ca_ channel gain-of-function is characterized by the release of ubiquitinated BK_Ca_ channels from the ER and their insertion into the membrane surface (Liu et al., 2014). Thus, impairing ubiquitination may lead to overexpression of BK_Ca_ channels relative to control conditions. Potential mechanisms underlying neuronal hyperexcitability following BK_Ca_ channels gain-of-function include: rapid AP repolarization that would favor reduced activation of SK_Ca_ and delayed rectifier K^+^ channels as well as facilitated the rate of recovery of Na_V_ channels from inactivation.

The mechanisms underlying the inhibitory and excitatory role of BK_Ca_ channels are complex (Figure 1). Functional studies have reported that the activation of BK_Ca_ channels is hyperpolarizing; thus the resulting net effect on membrane excitability is inhibitory. However, evidence suggests that the activation of BK_Ca_ channels can also facilitate high-frequency firing in some brain neurons, including CA1 pyramidal cells of the hippocampus (Gu et al., 2007). In physiological conditions, BK_Ca_ channels activate slowly during an AP, allowing intracellular Ca^2+^ to activate Ca^2+^-dependent conductances such as the small conductance Ca^2+^-activated K^+^ (SK_Ca_) channels, thereby inhibiting repetitive firing. The inhibitory effect following the activation of BK_Ca_ channels may result from a delay in the development of an AP spike or decrease in fAHP conductances. Altered extracellular K^+^ levels can modify the cell membrane potential to persistently depolarized values that may lead to paroxysmal discharges (Lebovitz, 1996). Interestingly, conversion from regular firing into burst firing upon the elevation of extracellular K^+^ has been observed in hippocampal slices (Jensen et al., 1994; Jensen and Yaari, 1997). Blockade of BK_Ca_ channels also can inhibit neuronal firing because the resulting AP broadening can allow the activation of slow-onset voltage-gated K^+^ channels, such as small SK_Ca_ channels and delayed rectifier K^+^ channels. The resulting K^+^ currents associated with an increased inactivation of voltage-gated Na^+^ (Na_V_) channels could slow the depolarization during an interspike interval. Further, excitation following the activation of upregulated BK_Ca_ channels may result from their role in the generation of fast spike repolarization and fAHP, which would favor a reduced activation of SK_Ca_ channels and delayed rectifier K^+^ channels and would indirectly facilitate the recovery of Na_V_ from inactivation (Gu et al., 2007). The upregulation of BK_Ca_ channels may cause large increase in extracellular K^+^, which in turn reduces the driving force for inhibitory K^+^ currents leading to enhanced neuronal excitability. The activation of BK_Ca_ channels can reduce neurotransmitter (GABA) release by shortening the duration of depolarization to allow Ca^2+^ entry via Ca_V_ channels, resulting in enhanced neuronal excitability (Hu et al., 2001; Raffaelli et al., 2004). There is also a possibility that the inhibitory and excitatory action of BK_Ca_ channels may be age dependent. Indeed, smaller BK_Ca_ channel currents were recorded in pyramidal neurons of the prefrontal cortex in developing animals compared with adolescent and adult animals (Ksiazek et al., 2013). Multiple lines of evidence indicate that a lower availability and/or expression of BK_Ca_ channels may contribute to the broadening of APs during repetitive firing (Shao et al., 1999; Faber and Sah, 2003). Therefore, the lower availability of BK_Ca_ channels in young animals may facilitate neuronal activity during this developmental stage. Given the relevance of BK_Ca_ channels in the control of neuronal excitability, these channels have been implicated in the pathophysiology of several neurological disorders associated with altered neuronal excitability, including seizure disorders.

## Bk_Ca_ channel loss-of-function hypothesis

### Bk_Ca_ channel loss-of-function and enhanced neuronal excitability in seizure disorders

Epilepsy consists of a group of chronic neurological disorders characterized by spontaneous and recurrent seizures. These seizures result from aberrant neuronal excitability associated with abnormal connections in the brain. Because the activation of BK_Ca_ channels limits the depolarization-induced bursting activity in neurons, it is assumed that a loss-of-function in BK_Ca_ channels will promote neuronal hyperexcitability, which can lead to seizures. Accordingly, reduced fAHP conductances were found in dentate gyrus granule cells obtained from patients suffering from temporal lobe epilepsy (Williamson et al., 1993). Similarly, idiopathic generalized epilepsy (mostly typical absence epilepsy) in humans has been associated with a single nucleotide deletion in exon 4 (delA750) of the *KCNMB3* gene encoding for BK_Ca_channel β 3 subunit (Lorenz et al., 2007). When expressed in a heterologous system, this mutation (BK_Ca_ channel β 3b-V4 subunit isoform) exhibited BK_Ca_ channel loss-of-function, characterized by fast inactivation kinetics (Hu et al., 2003). The mutated *KCNMB3* gene also has been found in patients with dup(3q) syndrome with seizures (Riazi et al., 1999).

BK_Ca_ channel loss-of-function has also been implicated in the pathophysiology of animal models of seizures and epilepsy. A transient loss of fAHP conductances was found in subicular neurons following a kindling model of epileptogenesis (Behr et al., 2000). In the genetically epilepsy-prone rat (GEPR), an inherited model of generalized tonic-clonic epilepsy, reduced fAHP conductances were reported in CA3 neurons of the hippocampus (Verma-Ahuja et al., 1995). Similarly, in preliminary experiments, we found that the current density of BK_Ca_ channels is significantly reduced in inferior colliculus (IC) neurons, the site of seizure initiation in this model. However, no significant change was observed in the abundance of BK_Ca_ channel α subunit proteins in IC neurons of the GEPR (N'Gouemo et al., 2009). Similarly, the expression of BK_Ca_ channel α subunit was not altered in the dentate gyrus of the Krushinskii-Molodkina rat, a model of inherited epilepsy (Savina et al., 2014). Nevertheless, the protein expression of BK_Ca_ channel β 4 subunits was elevated in the dentate gyrus of the Krushinskii-Moslodkina rat (Savina et al., 2014). The upregulation of β 4 subunit is consistent with loss-of-function because this subunit inhibits BK_Ca_ channel activity (Brenner et al., 2005). In a model of alcohol withdrawal seizures, BK_Ca_ channel loss-of-function was reported and characterized by reduced current density, decreased channel conductance and lower protein abundance of BK_Ca_ channel α subunit in IC neurons (N'Gouemo and Morad, 2014). However, these changes outlasted the finite period of alcohol withdrawal seizure susceptibility, suggesting that BK_Ca_ channel loss-of-function in IC neurons was associated with the long-term effects of alcohol withdrawal hyperexcitability. Whether BK_Ca_ channels in IC neurons play an important role in the pathogenesis of alcohol withdrawal seizures remains to be determined. In a pilocarpine post-status epilepticus model, a downregulation of BK_Ca_ channel α subunit mRNA and protein was found in the cortex and hippocampus, consistent with a loss-of-function of BK_Ca_ channels associated with seizure generation (Pacheco Otalora et al., 2008; Ermolinsky et al., 2011). Further analysis revealed that the remaining BK_Ca_ channels in the dentate gurus were essentially made of the BK_Ca_ channel STREX splice variant instead of the ZERO variant (Ermolinsky et al., 2011). Interestingly, inserting the STREX splice variant shifts the conductance/voltage relation of BK_Ca_ channels to the left so that the channels are active at more physiological Ca^2+^ and voltage levels (Shipston, 2013). However, elevated intracellular Ca^2+^ is associated with seizure activity and epileptogenesis (Sanabria et al., 2001; Raza et al., 2004), suggesting an altered function of the remaining STREX BK_Ca_ channels in the pilocarpine model.

### Bk_Ca_ channel loss-of-function and enhanced neuronal excitability in autism spectrum disorders

Autism spectrum disorders (ASD) are a heterogeneous group of genetic neurodevelopmental disorders characterized by impairment of social communication and behavioral problems. Interestingly, studies have reported a co-occurrence of ASD and epilepsy (Deykin and MacMahon, 1979). The prevalence of epilepsy and associated electroencephalogram abnormalities in ASD significantly exceeded that of the normal population (Tuchman and Rapin, 1997). The higher incidence of epileptiform electroencephalogram abnormalities was also reported in children with ASD without epilepsy (Tuchman and Rapin, 1997). Thus, autism may be classified as a disorder of neuronal excitability, suggesting a potential role for ion channels in the etiology of ASD. ASD-linked ion channels of interest include BK_Ca_ channels. A mutation in the *KCNAM1* gene, which encodes for the α subunit of BK_Ca_ channels, has been reported in some ASD patients with epilepsy (Laumonnier et al., 2006). The mutated *KCNAM1* gene also causes haploinsufficiency in ASD patients, suggesting a potential role of BK_Ca_ channels in the pathogenesis of ASD (Laumonnier et al., 2006). When expressed in a heterologous system, this mutation exhibits reduced BK_Ca_ channel currents consistent with a loss-of-function (Laumonnier et al., 2006). Whether the downregulation of BK_Ca_ channels directly contributes to the pathogenesis of autism-epilepsy phenotype remains unknown.

### Bk_Ca_ channel loss-of-function and reduced neuronal excitability in seizure disorders

Evidence shows that pharmacological blockade of BK_Ca_ channels can trigger seizures and status epilepticus, providing compelling evidence that BK_Ca_ channel loss-of-function can contribute to epileptogenesis (Young et al., 2003). However, mice lacking BK_Ca_ channel α (and β 1) subunits do not exhibit spontaneous seizures, consistent with no change or reduced CNS excitability (Sausbier et al., 2004). Thus, the elevated seizure susceptibility observed in animal models cannot be explained solely by a downregulation of BK_Ca_ channel α subunits. Notably, evidence shows that BK_Ca_ channels can be subjected to ubiquitination by CRL4A^CRBN^ and are therefore retained in the endoplasmic reticulum and prevented from trafficking to the cell surface. Deregulation of this control mechanism results in enhanced activity of neuronal BK_Ca_ channels and epileptogenesis (Liu et al., 2014). Notably, the cereblon (CRBN) co-localizes with BK_Ca_ channels in brain neurons and regulate their surface expression (Jo et al., 2005). The CRBN gene is highly expressed in the hippocampus, consistent with its role in the pathogenesis of limbic seizures (Liu et al., 2014).

## Bk_Ca_ channel gain-of-function hypothesis

### Bk_Ca_ channel gain-of-function and enhanced neuronal excitability in seizure disorders

Although BK_Ca_ channels are thought to reduce neuronal firing, evidence indicates that the gain-of-function of these channels can contribute to bursting activity and epileptogenesis. Indeed, upregulation of the α subunit and downregulation of the β 4 subunit of BK_Ca_ channels were found in the dentate gyrus neurons of Krushinskii-Molodkin rats subjected to audiogenic kindling, which induced enhanced seizure severity (Savina et al., 2014). These findings are consistent with the BK_Ca_ channel gain-of-function associated with enhanced seizure severity because the β 4 subunit inhibits BK_Ca_ channel activity. Notably, genetic deletion of the β 4 subunit of BK_Ca_ channels facilitates the development of pilocarpine-induced seizures that are associated with gain-of-function of BK_Ca_ channels, as characterized by elevated cell-surface expression of BK_Ca_ channels, enhanced Ca^2+^ sensitivity to BK_Ca_ channels, larger currents and high-frequency firing in the dentate gyrus of the hippocampus (Brenner et al., 2005; Shruti et al., 2012).

BK_Ca_ channel gain-of-function has also been found in human epilepsy. Accordingly, in a family of patients suffering from generalized epilepsy (mostly absence epilepsy) and paroxysmal dyskinesia, a missense mutation (D434G) in exon 10 of the *KCNMA1* gene that encodes the BK_Ca_ channel α subunit has been found (Du et al., 2005). When expressed in a heterologous system, this mutation gave rise to gain-of-function of BK_Ca_ channel currents characterized by larger currents, elevated open channel probability and enhanced Ca^2+^ sensitivity to BK_Ca_ channels (Du et al., 2005; Wang et al., 2009; Yang et al., 2010). The D434G mutation gain-of-function was potentiated in the presence of β 1, β 2, and β 4 subunits of BK_Ca_ channels (Díez-Sampedro et al., 2006; Lee and Cui, 2009). Notably, a polymorphism in the β 4 subunit has been associated with human epilepsy (Cavalleri et al., 2007). These findings suggest that D434G mutation-induced changes in BK_Ca_ channels contribute to neuronal hyperexcitability and lead to generalized seizures and paroxysmal dyskinesia.

### Bk_Ca_ channel gain-of-function and reduced neuronal excitability in seizure disorders

BK_Ca_ channels are found in excitatory neurons located in several brain sites, including the hippocampus, where they may promote high-frequency firing (Gu et al., 2007). Blockade of BK_Ca_ channels in these brain sites may reduce or suppress neuronal hyperexcitability. Consistent with this hypothesis, the blockade of BK_Ca_ channels suppressed pentylenetetrazole-induced epileptiform activity as well as spontaneous bursting activity in cortical neurons obtained from EL mouse, an inherited model of epilepsy (Jin et al., 2000). Similarly, picrotoxin-induced generalized tonic-clonic seizures give rise to BK_Ca_ channel gain-of-function characterized by elevated currents and high-frequency firing in somatosensory (barrel) cortical neurons of pre-sensitized animals (Shruti et al., 2008). Accordingly, the blockade of BK_Ca_ channels suppressed these picrotoxin-induced generalized tonic-clonic seizures (Sheehan et al., 2009). Thus, picrotoxin-induced seizure pre-sensitization may cause a maladaptive regulation (e.g., exit from the endoplasmic reticulum) of BK_Ca_ channels in brain neurons. In a fly model of ethanol intoxication/withdrawal, a blockade of *Slo1* gene neural promoter prevented the occurrence of ethanol-induced enhancement of electrographical seizure susceptibility, suggesting BK_Ca_ channel gain-of-function in the pathogenesis of alcohol withdrawal seizures (Ghezzi et al., 2012). However, this report raises some controversy with a rodent model of alcohol withdrawal seizures (N'Gouemo and Morad, 2014).

## Conclusion

The role of BK_Ca_ channels in the pathophysiology of diseases of neuronal excitability is complex, in part because the activity of these channels can be regulated by many metabolic factors that alter neuronal excitability, including phosphorylation and acidification. Compelling evidence suggests that BK_Ca_ channel loss-of-function and gain-of-function can both contribute to neuronal hyperexcitability that leads to enhanced seizure susceptibility. The identification of BK_Ca_ channel subunit mutations has been critical in determining the role of these channels in etiology and mechanisms for epileptogenesis and seizure generation, raising the possibility that BK_Ca_ channels may represent potential molecular targets for seizure suppression.

### Conflict of interest statement

The author declares that the research was conducted in the absence of any commercial or financial relationships that could be construed as a potential conflict of interest.
